# Statin use and non-melanoma skin cancer risk: a meta-analysis of randomized controlled trials and observational studies

**DOI:** 10.18632/oncotarget.20034

**Published:** 2017-08-08

**Authors:** Keming Yang, Andrew Marley, Huilin Tang, Yiqing Song, Jean Y. Tang, Jiali Han

**Affiliations:** ^1^ Department of Epidemiology, Richard M. Fairbanks School of Public Health, Indiana University, Indianapolis, IN 46202-2872, USA; ^2^ Department of Dermatology, Stanford University School of Medicine, Stanford, CA 94063, USA

**Keywords:** statins, non-melanoma skin cancer, meta-analysis

## Abstract

**Background:**

Existing evidence of the association between statin use and non-melanoma skin cancer (NMSC) risk has been inconsistent.

**Objective:**

To maximize statistical power to synthesize prospective evidence on this relationship.

**Materials and Methods:**

PubMed, EMBASE, Web of Science, Cochrane Central Register of Controlled Trials, and ClinicalTrial.gov were systematically searched up to December 11, 2016. A random-effects meta-analysis was conducted to calculate summary estimates.

**Results:**

Our meta-analysis of 14 randomized controlled trials (RCTs) including 63,157 subjects showed no significant association between statin use and NMSC risk (RR = 1.09, 95%CI = 0.85–1.39). However, meta-analysis of four observational studies including 1,528,215 participants showed significantly increased risk of NMSC among statin users compared to non-users (RR = 1.11, 95%CI = 1.02–1.22). Furthermore, ever using lipophilic statins (RR = 1.14, 95%CI = 1.04–1.24) or lower-potency statins (RR = 1.14, 95%CI = 1.03–1.26), as well as usage of any statin longer than one year (RR = 1.14, 95%CI = 1.09–1.18) were significantly associated with increased NMSC risk based on observational studies.

**Conclusions:**

Evidence from observational studies supported an association between statin use and increased NMSC risk. This finding should be interpreted with caution due to modest number of included studies, possible between-study heterogeneity and inherent limitations of observational studies.

## INTRODUCTION

Non-melanoma skin cancer (NMSC), including basal cell carcinoma (BCC) and squamous cell carcinoma (SCC) of the skin, is the most common cancer in the United States [1, 2]. Statins (HMG-CoA reductase inhibitors), potent cholesterol-lowering medications, have proven effective in the prevention of adverse cardiovascular events. Moreover, some studies also indicated a potential chemopreventive action of statins against overall cancer risk [3], as well as risks of cancers such as gastric [4], colorectal [5], breast [6], and prostate cancers [7]. However, evidence for risk of NMSC has been conflicting. Compared to controls, more NMSC cases were observed among the treatment group in some large randomized controlled trials (RCTs) of statins such as the Scandinavian Simvastatin Survival Study (4S) [8] and the Heart Protection Study (HPS) [9], but not others [10–12]. One previous meta-analysis found no significant association between statins and skin cancer [13]. A few prospective observational studies have specifically examined this hypothesis. Recently, Wang et al. [14] found that statins were associated with increased NMSC risk in the Women’s Health Initiative (WHI) cohort. A nested case-control study using Denmark national registries data also identified a significant association between statin use and slightly increased risk of BCC but not SCC [15]. Therefore, to maximize statistical power to synthesize prospective evidence on the relationship between statin use and NMSC risk, we conducted this meta-analysis of all eligible prospective observational studies and randomized controlled trials.

## RESULTS

### Study selection and study characteristics

**Figure 1:**
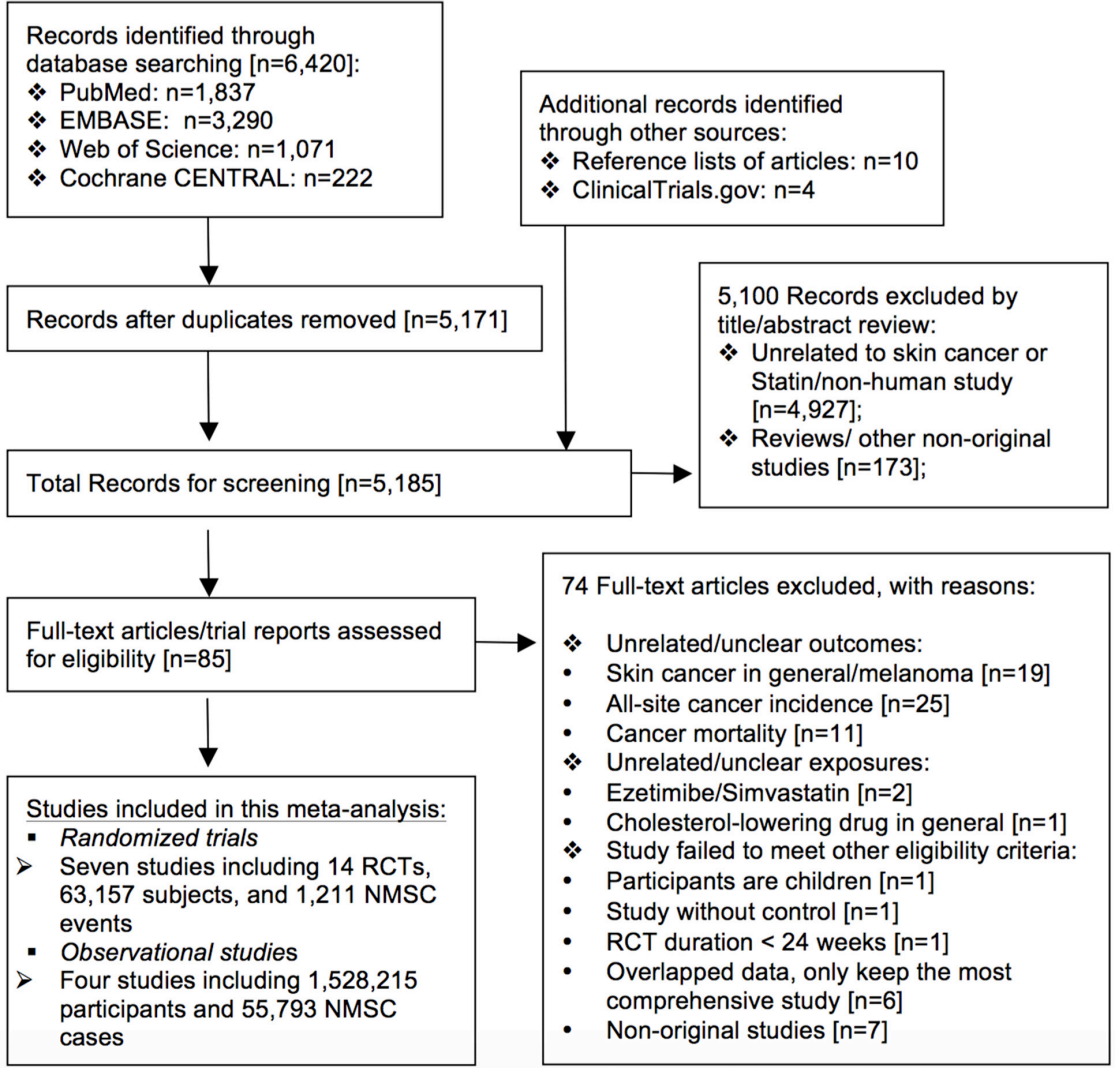
Flow chart of study selection

Among the 6,420 citations retrieved from the electronic databases, a total of seven reports that covered 14 RCTs including 63,157 subjects and 1,211 NMSC events [8–12, 16, 17], as well as four observational studies including 1,528,215 participants and 55,793 NMSC cases [14, 15, 18, 19] met our eligibility criteria and were included in this meta-analysis. The mean duration of included RCTs varied from 2 to 5.4 years while the mean/median follow-up time of included cohort studies varied from 3.5 to 10.5 years. A diagram of the study selection process is shown in Figure 1; basic characteristics of all included studies are shown in Table 1, and quality assessment results are shown in Supplementary Table 1.

**Table 1:** Characteristics of included studies in this meta-analysis

Randomized trials										
Report year	Trial Name	Study Design	Study Location	Mean Duration of exposure	Statins type (dose)	Treatment (NMSC cases/participants)	Control (NMSC cases /participants)	Age##	Men	White
1994	4S	RCT	5 European countries^#^	5.4 years	S (20–40 mg/day)	13/2221	6/2223	35–70	0.81	NR
2000	GISSI	RCT	Italy	2 years	P (20 mg /day)	1/2138	1/2133	C: 60.0 ± 10.4, T: 59.7 ± 10.4	0.86	NR
2001	AFCAPS /TexCAPS	RCT	USA	5.2 years	L (20–40 mg/day)	250/3304	243/3301	45–73	0.85	NR
2005	HPS	RCT	UK	5.3 years	S (40 mg/day)	243/10269	202/10267	64.0 ± 8.4	0.75	NR
2006	ALERT,FLARE,LCAS,LIPS,LiSA,NDA95 study 1–3	Pooled analysis of 8 RCTs	Variable	2.4 years	F (20–80 mg/day)	103/3512	125/3289	C: 55.8 ± 11.0, T: 55.1 ± 11.3	0.72	0.96
2011	JUPITER	RCT	USA	2 years	R (20mg/day)	15/8869	5/8864	66.1 ± 7.72	0.62	NR
2014	AURORA	RCT	Europe, America, Asia	2.4 years	R (10mg/day)	1/1389	3/1378	64.2 ± 8.6	0.62	0.85

Observational study (cohort and nested case-control studies)												
Publication year	First Author	Study Design	Study Location (data source)	Follow-up time	Exposure ascertainment	Outcome assessment	Statins type	Sample size	Adjusted RR (any use vs. no use)	Age	Men	**White**
2016	Wang	Cohort	USA (WHI OS+CT)*	Mean: 10.5 yrs	Self-report (medication containers)	Self–report	S,L,P,F,A,C,R	118,357	1.21 (1.07–1.35)	50–79	0	1
2015	Arnspang	Nest case-control	Denmark (Nationwide registries)	≥ 1 year^∆^	Prescription registry	Cancer Registry	S,L,P,F,A,C,R	464,288	BCC: 1.09 (1.06–1.13); SCC: 1.01 (0.91–1.11)	NR	NR	∼1
2009	Haukka	Cohort-record linkage	Finland (Nationwide registries)	Mean: 8.8 yrs	Prescription registry	Cancer Registry	S,L,P,F,A,C,R	944,962	1.28 (1.16–1.41)	Median: 60	0.5	NR
2009	Dore	Cohort-record linkage	USA (VATTC trial)**	Median: 3.5 yrs	Pharmacy Benefits Management database	Physical examinations + biopsy	S,L,P,F,A	608	0.92 (0.73–1.16)	90% >60	0.98	0.99

Note: 1. Full name of trials: 4S: Scandinavian Simvastatin Survival Study; GISSI: Gruppo Italiano per lo Studio della Sopravvivenza nell’Infarto Miocardico(Italian group for the study of the survival of Myocardial Infarction); AFCAPS/TexCAPS: Air Force/Texas Coronary Atherosclerosis Prevention Study; HPS: Heart Protection Study; ALERT: Assessment of LEscol in Renal Transplantation; FLARE: FLuvastatin Angiographic REstenosis trial; LCAS: Lipoprotein and Cholesterol Atherosclerosis Study; LIPS: Lescol Intervention Prevention Study; LiSA: Lescol in Severe Atherosclerosis trial; NDA95: new drug application studies; JUPITER: The Justification for the Use of Statins in Prevention: An Intervention Trial Evaluating Rosuvastatin (JUPITER) Trial; AURORA: A Study to Evaluate the Use of Rosuvastatin in Subjects on Regular Hemodialysis: An Assessment of Survival and Cardiovascular Events; 2. Abbreviations of statins: F: Fluvastatin; L: Lovastatin; P: Pravastatin; S: Simvastatin; R: Rosuvastatin; A: Atorvastatin; C: Cerivastatin; 3. NR: not reported; # Five European countries: Denmark, Finland, Iceland, Norway, Sweden; ## Age was shown in range or mean ± standard deviation, C: control, T: treatment; * WHI OS+CT: Women’s Health Initiative (WHI) Observational Study (OS) and Clinical Trial (CT); ** VATTC: Veterans Affairs Topical Tretinoin Chemoprevention Trial; ^∆^: Incident NMSC during 2005–2009; Prescription: 1995 to 1 year before diagnosis; ∼: Approximately;

### Meta-analysis of statin use and NMSC risk

**Figure 2:**
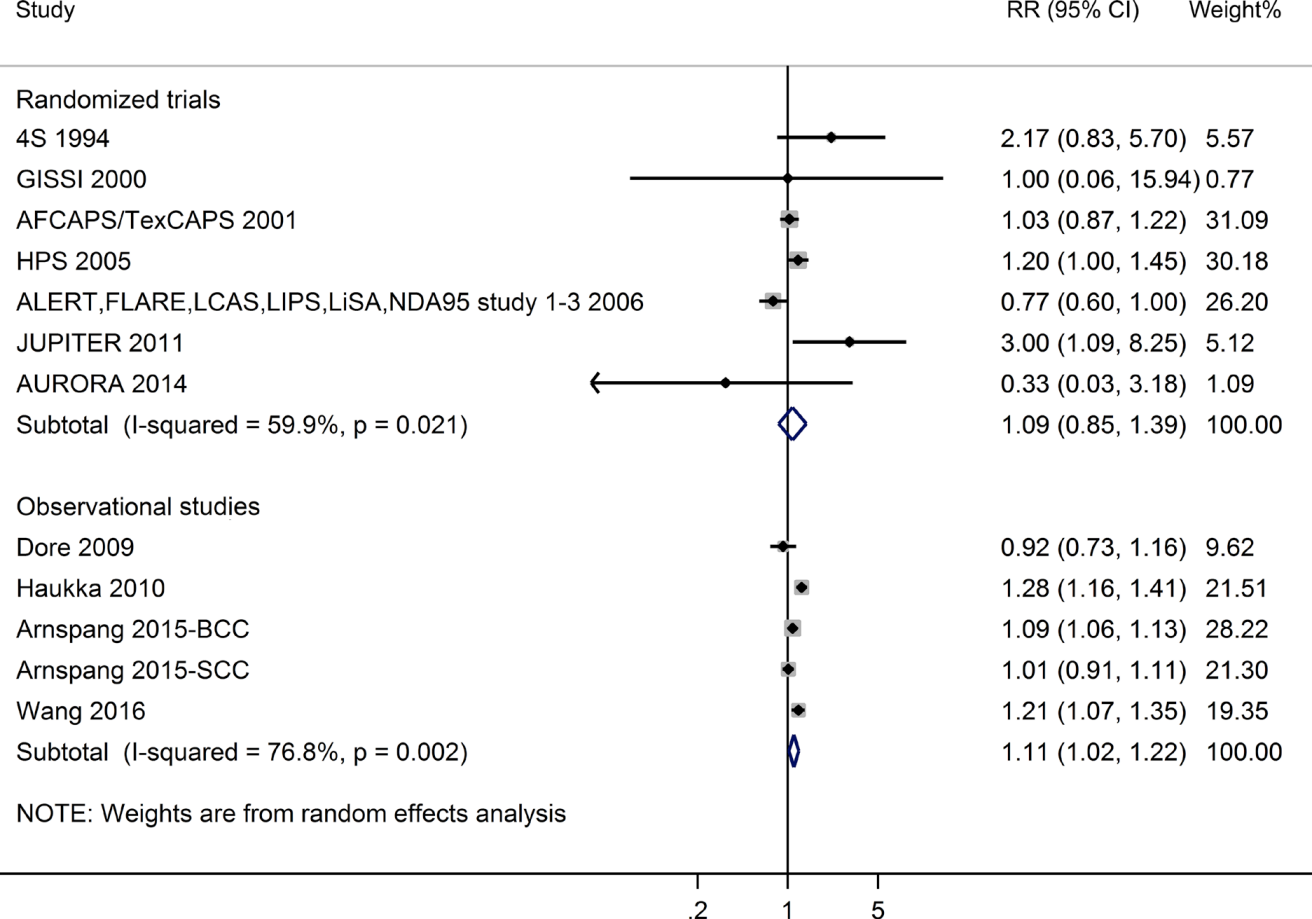
Forest plot: meta-analysis of statin use and non-melanoma skin cancer risk

Meta-analysis of RCTs did not show a significant association between statin use and NMSC risk (RR = 1.09, 95%CI = 0.85–1.39; I^2^ = 59.9%; *P* for heterogeneity = 0.02). However, the meta-analysis of observational studies revealed a significant association. Statin users have a higher risk of NMSC than non-users (RR = 1.11, 95% CI = 1.02–1.22; I^2^ = 76.8%; *P* for heterogeneity = 0.002) (See Figure 2). Begg’s and Egger’s tests showed no significant evidence of publication bias (meta-analysis of RCTs: *P* for Begg’s test = 0.88, *P* for Egger’s test = 0.70; meta-analysis of observational studies: *P* for Begg’s test = 0.62, *P* for Egger’s test = 0.84).

### Subgroup analysis of statin use and NMSC risk

The results of subgroup analyses are shown in Table 2. Between-group heterogeneity was not statistically significant in most subgroups (*P* > 0.05). None of the subgroup analyses detected a significant association based on RCTs (*P* > 0.05). However, subgroup analyses of observational studies revealed significantly increased risks of NMSC among ever-users of lipophilic statins (RR = 1.14, 95% CI = 1.04–1.24) or statins with lower potency (RR = 1.14, 95% CI = 1.03–1.26), compared to never-users. Additionally, users of statins for more than one year had significantly higher NMSC risk than never-users (RR = 1.14, 95% CI = 1.09–1.18).

**Table 2:** Meta-analysis and subgroup analysis of randomized trials and observational studies

		RCTs				Observational Studies			
	Subgroup analyses	No. of studies	RR (95%CI)	Heterogeneity		No. of studies	RR (95%CI)	Heterogeneity	
				**I**^2^	*P*			**I**^2^	*P*
Total		7	1.09 (0.85–1.39)	59.9%	0.02	4	1.11 (1.02–1.22)	76.8%	0.002
By Category	Lipophilic	4 (s,f,l)	1.04 (0.82–1.31)	69.5%	0.02	2 (s,f,l.a)	1.14 (1.04–1.24)	36.5%	0.10
	Hydrophilic	3 (r,p)	1.39 (0.35–5.55)	39.1%	0.19	2 (p)	0.96 (0.81–1.15)	0.0%	0.70
By Potency	Lower	5 (f,l.s.p)	1.04 (0.83–1.30)	57.3%	0.05	2 (f,l.s.p)	1.14 (1.03–1.26)	39.0%	0.08
	Higher	2 (r)	1.27 (0.15–10.46)	67.1%	0.08	2 (a)	1.05 (0.94–1.17)	0.0%	0.55
By Duration	< 5 years	4	1.08 (0.43–2.71)	58.2%	0.07	3	< 1 year: 1.08 (0.99–1.18)	39.0%	0.16
						3	≥ 1 year: 1.14 (1.09–1.18)	30.8%	0.13
						3	1–< 3 years: 1.14 (1.04–1.25)	47.9%	0.10
						3	3–< 5 years: 1.14 (1.08–1.20)	0.0%	0.67
	≥ 5 years	3	1.14 (0.94–1.37)	40.4%	0.19	3	≥ 5 years: 1.16 (1.04–1.29)	55.7%	0.08

Note: s: Simvastain, f: Fluvastain, l: Lovastatin, r: Rosuvastatin, p: Pravastatin, a: Atorvastatin; Comparisons were conducted between any use and no use of statins (or statins of different type);

## DISCUSSION

Our meta-analysis of four prospective observational studies involving 1,528,215 participants and 55,793 NMSC cases suggested a significant and positive association between statins and NMSC risk. However, our meta-analysis of 14 RCTs with 63,157 participants and 1,211 NMSC events did not observe such an association. This may be due to the fact that RCTs usually have shorter follow-up period of time than observational studies and an increased risk of rare events (e.g. cancers) may not be detected during a relatively short duration. Besides, cancer events are not the primary endpoints of the RCTs for statins. Therefore, observational studies may have some advantage of examining a long-term drug-safety issue.

One previous meta-analysis evaluated statins and all-cancer risk, among which only four RCTs were related to NMSC, and their pooled results indicated a significant association between statins and increased NMSC risk [20]. Another meta-analysis conducted by Li et al. [13] found no significant association between statins and risk of either melanoma or NMSC. However, that study also included skin cancer cases of unspecified type in the meta-analysis of NMSC. To our knowledge, ours is the first meta-analysis specifically examining the relationship between the risk of NMSC and statins, as well as statins of different potency, category, and duration of use.

Melanoma, SCC, and BCC are three major types of skin cancer. The underlying mechanisms of melanoma and non-melanoma skin cancers (SCC and BCC) are not the same. In terms of melanoma, which is the most serious type of skin cancer, most randomized controlled trials and observational studies published so far showed non-significant association between statin use and melanoma risk [13]. Recently, sixteen years of follow-up of the Long-term Intervention with Pravastatin in Ischemic Disease (LIPID) trial also reported no excess cases of melanoma in the intervention group, compared to the control group [21].

We still lack substantial understanding of the biological mechanisms by which statins affect cancer risk, and whether specific properties of statins make any difference in this process. Some studies have indicated that the immunomodulatory effects of statins may be responsible for its potential carcinogenic actions. A statin-induced increase in CD4+CD25+ regulatory T cells (Tregs) may impair the host antitumor immune response through suppressing the tumor specific effector T cell response, which may lead to an increased risk of cancer [22]. Notably, immunosuppression is an emerging risk factor for NMSC [23], and may explain the high incidence of NMSC in the elderly populations who are susceptible to immunosenescence. In addition, immunosuppressant therapies constitute a major risk factor of developing post-transplantation skin cancers among organ transplant recipients [24]. In kidney transplant recipients, high Tregs levels within the peripheral circulation were associated with new SCC development [25]. On the contrary, some research has also suggested that statins exert a protective action against skin cancers by inhibiting the mevalonate and hedgehog signaling pathways, disrupting cancer cell growth and inducing keratinocyte apoptosis [26]. As statins have also been associated with reduced risks of several other types of cancers [7, 20], it is possible that statins may play roles in affecting different cancer risks through various biological pathways.

Our findings should be interpreted with caution due to the modest number of studies we were able to include, possible between-study heterogeneity, as well as the residual confounding inherent in observational studies. Due to the unavailability of original data, we could not conduct meta-analysis separately for BCC and SCC. We also could not estimate dose-response effects as available data were too heterogeneous to pool.

In conclusion, our meta-analysis of observational studies supported an association between statin use and increased NMSC risk. In future, ongoing post-marketing surveillance and long-term follow-up studies of RCTs could provide ongoing monitoring of new NMSC cases that may be related to the use of statins. More prospective studies with large sample sizes could be conducted to further evaluate the association between statins and NMSC risk. In addition, basic research on cholesterol metabolism and tumor formation is needed to explore underlying mechanisms.

## MATERIALS AND METHODS

This meta-analysis was conducted according to the Preferred Reporting Items for Systematic reviews and Meta-analyses (PRISMA) guidelines [27].

### Systematic search

We conducted a comprehensive and systematical literature search in PubMed, EMBASE, Web of science, Cochrane Central Register of Controlled Trials (CENTRAL), and ClinicalTrials.gov registry to identify all prospective evidence (all from inception to December 11^th^, 2016 with no language limitation). References list from all relevant review articles, meta-analysis, and the identified articles were manually checked. The following terms were used for literature search: ((hydroxymethylglutaryl coenzyme A reductase inhibitor) OR (hydroxymethylglutaryl-coa reductase inhibitors) OR (HMG-CoA reductase inhibitors) OR (statins) OR (atorvastatin) OR (fluvastatin) OR (lovastatin) OR (mevastatin) OR (pravastatin) OR (pitavastatin) OR (rosuvastatin) OR (simvastatin) OR (cerivastatin)) AND ((melanoma) OR (non-melanoma) OR (nonmelanoma) OR (basal cell carcinoma) OR (squamous cell carcinoma) OR (cancer) OR (neoplasms) OR (neoplasm) OR (skin cancer)).

### Eligibility criteria

Both randomized controlled trials and prospective observational studies (cohort study and nested case-control study) were considered. Included studies should meet the following criteria: (1) Published and unpublished RCTs of statins that reported NMSC cases as adverse events (length of trial ≥ 24 weeks); (2) Cohort or nested case-control studies that examined the association between statin use and NMSC incidence; (3) All study participants were adults aged ≥ 18 years. NMSC cases referred only to SCC and BCC in this meta-analysis. Retrospective case-control studies were excluded as they were subjected to various biases. Also, to minimize bias, we used safety data from only the original trials rather than prolonged post-trial follow-up (if available), as the majority of all subjects received open-label lipid-lowering treatment during the latter. Studies did not provide sufficient information or failed to meet eligibility criteria were also excluded.

### Data extraction

Two reviewers (K.Y. and A.M.) independently performed the study selection, data extraction, and quality assessment. Numbers of NMSC cases were extracted from randomized trials and adjusted risk ratios were extracted from observational studies for data synthesis. As two included RCTs did not report NMSC events in the manuscript (JUPITER and AURORA) [11, 16], the case numbers were extracted from the “Serious Adverse Events” section on the ClinicalTrials.gov registry website. Study characteristics including first author’s name, publication/report year, trial name, study design, study location, sample size, study length, type of statins, exposure and outcome assessment, as well as population characteristics including age, sex and race of the study population in each included study were also extracted.

### Quality assessment

The quality of randomized trials was evaluated by Cochrane Risk of Bias (RoB) Tool [28]. We used Cochrane RoB tool to assess potential study bias in 6 domains, including random sequence generation (selection bias), allocation concealment (selection bias), blinding of participants and personnel (performance bias), blinding of outcome assessment (detection bias), incomplete outcome data (attrition bias), and selective reporting (reporting bias). The quality of included observational studies was assessed by Newcastle-Ottawa quality assessment scale (NOS) [29]. Each cohort/case-control study was evaluated on three broad perspectives: the selection of the study groups, the comparability of the groups, and the ascertainment of the exposure and the outcome of interest.

### Data synthesis and further analysis

We used the random-effects model (DerSimonian and Laird inverse variance method) to estimate summary relative risk (RR) with a 95% confidence interval (95% CI). I^2^ statistics were used to assess between-study heterogeneity. Additionally, subgroup analyses were separately performed for RCTs and observational studies to explore the source of heterogeneity, based on: (1) category of statins (Lipophilic: Fluvastatin, Lovastatin, Simvastatin, Atorvastatin; Hydrophilic: Pravastatin, Rosuvastatin); (2) potency of statins (Lower: Fluvastatin, Lovastatin, Pravastatin, Simvastatin; Higher: Atorvastatin, Rosuvastatin); and (3) duration of statin use (< 1 year vs. ≥ 1 year; < 5 years vs. ≥ 5 years). Publication bias was examined using Begg’s and Egger’s tests. All statistical analyses were performed with STATA version 14.2. Two-sided α ≤ 0.05 was the significance level.

## SUPPLEMENTARY MATERIALS TABLE





## Abbreviations

NMSC: non-melanoma skin cancer
BCC: basal cell carcinoma
SCC: squamous cell carcinoma
RCT: randomized controlled trial
RR: risk ratio
CI: confidence interval
