# Supplementary material for: Statin use and non-melanoma skin cancer risk: a meta-analysis of randomized controlled trials and observational studies
Source: Oncotarget. 2017 Aug 8;8(43):75411–7. doi: 10.18632/oncotarget.20034 (PMC5650431; doi:10.18632/oncotarget.20034)
Supplement: Supplementary file 1 [file oncotarget-08-75411-s001.pdf]

Statin use and non-melanoma skin cancer risk: a meta-analysis of randomized controlled trials and observational studies

SUPPLEMENTARY MATERIALS

Supplementary Table 1: Risk of bias and quality assessment of included randomized trials and observational studies. See Supplementary_Table_1
