# Supplementary material for: Statin use and non-melanoma skin cancer risk: a meta-analysis of randomized controlled trials and observational studies
Source: Oncotarget. 2017 Aug 8;8(43):75411–7. doi: 10.18632/oncotarget.20034 (PMC5650431; doi:10.18632/oncotarget.20034)
Supplement: Supplementary file 2 [file oncotarget-08-75411-s002.docx]

**Supplementary Table 1: Risk of bias and quality assessment of included randomized trials and observational studies**

| **Randomized trials** | | | | | | | | | | | | | | | | |
| --- | --- | --- | --- | --- | --- | --- | --- | --- | --- | --- | --- | --- | --- | --- | --- | --- |
| **Study** | **Cochrane Risk of Bias (RoB) Tool** | | | | | | | | | | | | | | **Overall** **Quality** | |
|  | **Selection bias** | | **Selection bias** | | | | **Performance bias** | | **Detection bias** | | **Attrition bias** | | **Reporting bias** | |  |  |
|  | Random sequence generation | | Allocation concealment | | | | Blinding of participants/personnel | | Blinding of outcome assessment | | Incomplete outcome data | | Selective reporting | |  |  |
| 1994 4S | low | | low | | | | low | | low | | low | | low | | High | |
| 2000 GISSI | low | | unclear | | | | high | | high | | low | | low | | Medium | |
| 2001 AFCAPS | low | | unclear | | | | low | | low | | unclear | | low | | Medium | |
| 2005 HPS | low | | low | | | | low | | unclear | | low | | low | | High | |
| 2011 AURORA | low | | unclear | | | | low | | unclear | | high | | low | | Medium | |
| 2014 JUPITER | low | | unclear | | | | low | | low | | low | | low | | High | |
| **Cohort study- Newcastle-Ottawa quality assessment scale (NOS)** | | | | | | | | | | | | | | | | |
| **Study** | **Selection** | | | | | | | **Comparability** | | **Outcome** | | | | | | **Overall**  **Quality** |
|  | Representativeness of the exposed cohort | Selection of the non-exposed cohort | | | Ascertainment of exposure | Outcome of interest not present at start of the study | | Comparability of cohorts on the basis of the design or analysis | | Assessment of outcome | Follow-up long enough for outcomes to occur | | | Adequacy of follow up of cohorts | |  |
| 2009 Haukka | + | + | | | + | + | | ++ | | + | + | | | + | | High |
| 2009 Dore | + | + | | | + | + | | ++ | | + | + | | | + | | High |
| 2016 Wang | + | + | | | + | + | | ++ | | 0 | + | | | + | | High |
| **Nested case-control study- Newcastle-Ottawa quality assessment scale (NOS)** | | | | | | | | | | | | | | | | |
| **Study** | **Selection** | | | | | | | **Comparability** | | **Exposure** | | | | | | **Overall**  **Quality** |
|  | Case definition | Representativeness of cases | | Selection of controls | | Definition of controls | | Comparability of cases and controls | | Exposure ascertainment | | Same methods for case/control | | Non-response rate | |  |
| 2015 Arnspang | + | + | | + | | + | | ++ | | + | | + | | + | | High |

Note: 1. Low: low risk of bias; High: high risk of bias; unclear: unclear risk of bias; 2. NOS scale: A study can be awarded a maximum of one ‘+’ for each numbered item within the Selection and Exposure/Outcome categories. A maximum of two ‘++’ can be given for Comparability;
